# Usability, acceptance, and the role of realism in virtual humans for breathing exercise training

**DOI:** 10.1038/s41598-024-82886-7

**Published:** 2025-01-09

**Authors:** Sanobar Dar, Aniko Ekart, Ulysses Bernardet

**Affiliations:** College of Engineering and Physical Sciences, Aston University, Birmingham, B4 7ET UK

**Keywords:** Breathing, Breathing exercises, Relaxation, Virtual reality, Virtual humans, Intelligent agents, Healthcare technology., Computer science, Rehabilitation, Occupational health, Quality of life

## Abstract

Breathing exercises are used to address a multitude of issues ranging from mental health to physical well-being. While they are recommended for various health conditions, access to professional support is often limited due to time and cost constraints. Virtual Reality technology offers a promising solution for enhancing mindfulness and relaxation practices, including breathing exercises. In our study, we explored the effectiveness of a computer-generated “virtual breathing coach”. Our virtual coach has a human appearance, speaks in a human voice, and displays breathing physiology while guiding the participants through a set of breathing exercises of different cycle lengths. The agent exhibits breathing behaviour with the aim of augmenting the influence the agent exerts through the mechanisms of physiological entrainment known from human-to-human interaction. Our empirical, exploratory study with 20 participants, was conducted using non-immersive VR where the system ran on a web browser. On the one hand, we investigated the perception of the virtual human coach, and on the other hand, we evaluated the usability, acceptability, and adherence of the system. We also evaluated the role of realism in acceptance of and adherence to the virtual coach. Participants’ responses were recorded using questionnaires. Participants were influenced by the virtual coach and adhered to it if they perceived the virtual coach as more realistic. The participants reported that the system ran well, and they would be willing to continue using the system in the future. Although the virtual human-to-human interaction experience provided by our system is not on par with the naturalistic human-to-human interaction, the results indicate that people are willing to replace a real human coach with a virtual coach. Systems like this are a step towards improving the affordability and on-demand access to another human, such as a coach in our system, especially where access and affordability are limited such as in healthcare. The qualities such as trust and likeability in an embodied interaction can make this interaction more efficient and effective.

**Supplementary Information:**

The online version contains supplementary material available at 10.1038/s41598-024-82886-7.

## Introduction

The Coronavirus (COVID-19) pandemic has affected a large number of people worldwide. Not only does the SARS-CoV-2 virus cause respiratory infections, but it is also associated with long-term effects of anxiety and depression^1^. While there are several medications for treating both, physiological and psychological effects, one of the simplest and most widely used treatments is the use of breathing exercises^2^. The use of breathing exercises has the advantage of being affordable, unintrusive, and easy to use. Due to the bidirectional relationship of breathing with emotion and cognition^3^, improving breathing has a positive effect on people’s physical and mental health^4^. Breathing exercises have been shown to regulate breathing, relieve anxiety, improve symptoms of dyspnoea, and improve quality of life^5^. A breathing exercise regime prescribed by a breathing therapist can encourage a patient to regulate their breathing. A therapist not only provides technical guidance in a proficient and systematic approach but also provides a sense of interpersonal connection which is often a key facilitator for successful therapy^6^. At the mechanistic level, this connection is established through the exchange of implicit nonverbal cues in addition to verbal instructions.

During situations like the pandemic, limited access to therapists can prove counter-productive, thereby causing high attrition rates in healthcare interventions. People are concerned about the accessibility to healthcare due to its high cost and unavailability of trained experts, and the worsening of their health conditions^7^. A large number of people, therefore, resort to online platforms for help, such as self-help videos and exercises including self-help breathing exercises^8^. Although the latter are readily available for people to use without any supervision^9^, adhering to such self-help routines over a longer period is often challenging. Tele-healthcare provides marginal support in terms of accessibility, where tele health is defined as “the delivery and facilitation of health and health-related services including medical care, provider and patient education, health information services, and self-care via telecommunications and digital communication technologies”^10^. But it suffers from similar limitations as any remote healthcare delivery^11^ such as long waits and limited availability.

In this paper, we present an online system where a virtual coach guides a user’s breathing through a set of established breathing exercises. An advantage of using a virtual human coach is that they are found to be helpful in user adherence in long-term interventions^12^. We aim to overcome the limitations of affordability and on-demand accessibility and at the same time provide a stimulating environment through a sense of human connection via verbal and nonverbal cues using our Virtual Human Breathing Coach. The context of the use of such a system is that users can use it in the comfort of their homes.

Our implementation of the virtual coach has a human appearance, human voice, co-speech gesticulation, and, importantly, displays breathing movements of the thorax and head. The virtual coach guides the user through a breathing cycle of inhale, hold breath, exhale, and hold breath. The breathing cycle lengths for inhale, hold, exhale, and hold vary between exercises to allow us to establish the optimal length of the breathing cycle for each user. This is especially important for the effectiveness of the exercise in situations such as relaxation. The manipulation of the cycle length not only allows investigation of the optimum cycle length of the breathing exercises but also help determine the ideal pace of the exercise. The breathing exercises are chosen for ease of use, adaptability, and effectiveness in realising relaxation. We conducted an empirical study with 20 participants using a 2D system that ran on a screen to answer the following research questions:

1) Do participants find the system usable?

2) Is the virtual coach effective in influencing the breathing of the participants?

3) What is the participants perception of the virtual coach?

4) What is the optimum frequency of the breathing exercise cycle?

### Background

Breathing in humans is a highly complex physiological process that not only affects the physical, but also mental health^4^. Patients with mild to severe breathing conditions are often prescribed predefined breathing exercises. Typically, a trained therapist gauges the breathing pattern of the patient to then teach the patient how to control their breathing and avoid breathing too fast or too slow. The patient follows the instructions of the therapist to achieve a state of controlled breathing through guided breathing. Among emerging technologies in healthcare^13–15^, Virtual Reality (VR) shows a lot of potential in the application for relaxation and mindfulness due to the ability of VR to alter users’ physiological responses^16–18^. An analysis shows that most of these are either text-based or use voice instructions to prescribe exercises to users, with a few that include video of an instructor or a virtual human as a coach to prescribe exercises to users^19^.

### Breathing exercises for relaxation

Regulating breathing can help improve physical and mental health^5,9,20^. Specific breathing techniques can have a positive effect on a person’s emotional state, thus helping improve their mental health^4^. During a working period, an increase in respiration frequency leads to increased oxygen consumption and a rise in carbon-dioxide production^21^. Our respiratory system, therefore, adapts and adjusts the breathing rhythm and volume to help meet the body’s demands. This demonstrates the sensitivity of respiration: relaxation has beneficial effects and stress has detrimental effects^22^. The use of breathing exercises has proven to be efficient in reducing anxiety, improving immunological parameters, and improving the endurance of the respiratory muscles and are therefore prescribed to patients having mild to severe breathing conditions such as asthma, stress, anxiety, insomnia, panic disorder, chronic obstructive pulmonary disease^23^. Such techniques work by altering the breathing rhythm in a way that breathlessness is reduced^24^. An example of a technique is “Yoga” breathing where the breathing is actively regulated to mentally calm down and prepare for concentration and meditation. This is based on a Sanskrit term - pranayama (prana: breath, vital energy; Ayama: extending, controlling)^25^. It is an effective technique for reducing arousal in treating patients with insomnia^24^. Physiotherapists have also successfully used the Papworth method, a technique that encourages gentler, more relaxed breathing using the abdomen rather than the chest^26^. It has been used for patients with asthma and dysfunctional breathing to control and correct breathing^27^. Another breathing relaxation technique that is used by soldiers to reduce stress, stay calm, and retain self-awareness is the so-called “Box breathing”^28^. Typically, in Box breathing, users inhale for 4 seconds, hold the breath for 4 seconds, then exhale for 4 seconds and lastly hold the breath for 4 seconds. Box breathing is one of the simplest and easiest to perform techniques that can be performed from the start without any supervision. Research suggests that the Box breathing technique can increase the efficiency of breathing by increasing the volume of air in the lungs and allowing them to better handle conditions with less oxygen, especially in situations like anxiety and prolonged sitting^23^.

### Digital breathing interventions

Delivery of healthcare using digital interventions is becoming increasingly popular especially in the form of breathing exercises to improve physical and mental well-being. There are several digital breathing interventions that are being used by people. Examples range from electronic devices that are used in guided breathing such as device guided breathing where a device sends audio triggers to encourage users’ to synchronise their breath to the guiding tunes^9^, to several breathing exercise apps, such as Headspace, Calm etc. Although, mobile apps offer an on-demand access compared to device dependent interventions, most of them lack the individualised regime which feedback offers. The lack of supervision and interactivity also makes it difficult to adhere to these techniques over a longer period. More interactive online platforms such as BrightBeat - which provides visual and auditory feedback to computer users to encourage more gentler breathing^29^or Just Breathe - an In-Car breathing intervention that uses haptic and voice-based guidance in the form of vibrations felt on the back of the user while seated in order to synchronise their inhales and the inhales with the vibrations^30^. The study showed positive results in slowing the breathing rate down. A trend of using VR technology in such systems is also observable such as - Life Tree where users practice pursed lip technique in VR with inhalation and exhalation corresponding to increase and decrease in the growth of trees within the VR environment respectively, offer a more personalised regime^31^or DEEP - a VR game that provides an immersive and relaxing experience using biofeedback^32^. A study of DEEP showed reduced levels of stress and anxiety in children.

### 3D Interactive systems and VR in healthcare

The use of Virtual Reality technology is effective in addressing many health-related issues such as anxiety, depression, schizophrenia, phobias, post-traumatic stress, substance misuse, and eating disorders^33–37^. With the help of technological developments in virtual reality, we can create environments that mimic natural settings and provide users with an experience that is close to a real experience. The reason Virtual Reality is being extensively explored in such applications is that, firstly, it allows full control over the environment the user is exposed to^38^and, secondly, it has the ability to provide a physiological perception of presence, i.e., the feeling of being immersed within the simulated environment where the user can recognise that the environment is not real, but they ideally behave and respond similarly to how they would in a real environment^39^. This allows laboratory studies, clinical interventions, or therapy sessions to be conducted in a controlled but realistic setting^40^. The advantage of creating realistic virtual environments is that it aids in immersion^41^where the feeling of immersion in the virtual reality environment has been shown to alter people’s complex physiological mechanisms such as their breathing rate^18^. This is useful in some of the above-mentioned health issues since improving breathing can have a positive effect on people’s physical and mental health^42^.

### Physical and virtual agents for healthcare and wellbeing

The use of physical and virtual agents as a tool in breathing interventions has also been explored in healthcare and wellbeing. Robots in the form of physical agents such as Omnie uses audio, verbal, and haptic cues by shrinking and expanding which supports deep breathing to reduce anxiety^43^. Similar examples are CALM Robot^44^and CAKNA^45^. Like Omnie, CALM robot uses tactile sense to guide users to a slow breathing and CAKNA which was shown to reduce anxiety by focusing on breathing.

Closely related to humanoid robots are computer-generated autonomous “virtual humans”, that can be regarded as “a form of human-machine interface where the machine is communicating with the user through the representation of an artificially intelligent animated human form.”^46^. Virtual humans are used as teachers, advisors, social companions, and increasingly, as coaches^47^. Virtual human technology offers on-demand access for users in the comfort of their homes, and correspondingly, virtual humans as coaches in healthcare is an emerging application domain^48^. The interaction with virtual human’s ranges from being a one-way communication process to a closed loop, two-way, multi-channel, multi-modal interaction^49^. Studies have shown that even in simple, one-way communication systems the expressed personality traits of a virtual coach can have a significant effect on learners’ experience^50^. Even a cartoonish 2D virtual coach face with limited nonverbal gestures used for guided meditation training was found effective in increasing the frequency of practice, and capable of providing a more effective training and coaching than a self-administered program using written and audio materials^51^. Similar to this is Autotutor- a real-time adaptive system adapts its instructions based on a real-time assessment of a student’s affective state. Significant learning improvement was reported for students who worked with the Autotutor^52^. A 2D virtual agent has been used as a meditation coach. It gives breathing instructions to the participants to induce a relaxed state^19^and also reads the breathing of the user and gives breathing instructions based on the user’s breathing in a close loop interaction^18^. The virtual coach was further found useful in alleviating chronic pain and stress using yoga and meditation^53^. Another example of a closed-loop interaction is where both the user and the virtual human get into a state of physiological synchrony^54^.

While there are systems that harness a naturalistic closed-loop interaction between the user and the virtual human, the use of a realistic virtual human as a coach is still an emerging improvement. Most of the systems use a flat 2D cartoon character face or body with little or no gesticulations and as such are limited in their ability to instil a sense of personal connection contributing to a real-life like interaction between the human and the virtual agent where the agent is able to simulate the key social aspects of human-to-human interaction. While the uncanny valley effect presents a significant challenge because it can cause discomfort and unease in users when they interact with virtual humans that are almost, but not quite, lifelike^55^, ongoing advancements in technology are gradually mitigating its impact. Researchers and developers are continuously working to enhance the realism and emotional expressiveness of virtual humans. By improving gestures, facial animations, body language, and voice synthesis, they aim to create virtual humans that can interact in ways that feel more natural and less unsettling to users. Moreover, the integration of sophisticated artificial intelligence allows these virtual humans to better understand and respond to human emotions, making interactions smoother and more intuitive. This progress not only helps in reducing the discomfort associated with the uncanny valley but also opens new possibilities for their application in various fields^56^. For instance, in healthcare, more lifelike virtual humans can provide patients with a sense of comfort and familiarity, which is crucial for effective therapy and support. As technology continues to evolve, the gap between virtual and real humans will narrow, making the benefits of using realistic virtual humans increasingly outweigh the drawbacks. This ongoing improvement ensures that virtual humans will become more accepted and integrated into our daily lives, providing valuable services and enriching our interactions. This is further supported by research suggesting that a more anthropomorphised agent - be that physical anthropomorphism of the character, of the physical space they are placed in or the non-physical anthropomorphism such as displaying emotions using subtle nonverbal cues, is preferred by people^57–59^. One of the reasons for a preference of realistic agents might be that human-to-human interaction is a sophisticated process that, for example, allows to accurately identify and distinguish each other’s emotions in a given situation. At the psychological level this taps into the mechanisms of “Theory of Mind”, i.e. the capability of humans to understand that people’s thoughts, beliefs, emotions, etc. can differ from each other^60^. This mechanism allows us to attribute mental states to others and make inferences about others emotional state based on their body language, facial expressions, tone of voice, etc^61^. , enabling us to navigate social interactions, understand others, and respond empathetically, contributing to an overall affective interaction. Therefore, realistic virtual humans that accurately replicate these cues might facilitate affective communication, conveying emotions through nonverbal clues, leading to a sense of personal rapport.

In summary, empirical evidence supports the use of a realistic virtual human in a healthcare domain. While previous interventions have shown a positive effect on breathing rate, the added value that our system is providing is the use of a realistic 3D virtual human as a breathing coach. Unlike systems that do not use a humanoid agent in the form of virtual human or robot, the humanoid virtual coach can facilitate rapport building^62,63^and provide a stimulating environment where the user can get a sense of personal connection^64^ where users can get influenced from the virtual coach as they do from a real human coach. These unique opportunities are only possible when we use a realistic virtual coach capable of verbal as well as nonverbal interactional skills instead of a 2D character. Hence, the contribution of our work lies in using a realistic 3D, humanoid digital avatar with verbal and nonverbal cues, that can be used in the comfort of our homes for guided breathing.

### System framework

The Virtual Breathing Coach is a relaxation system where a virtual human coach guides users’ breathing and influences them to attain relaxation through guided breathing. The virtual human coach guides users along a set of Box breathing exercises that vary in difficulty level. The implementation of the system allows it to run standalone in any web browser and is deployed such that it works seamlessly in the browser without the need for any installation or instrumentation.

### Design of the virtual coach

**Fig. 1:**
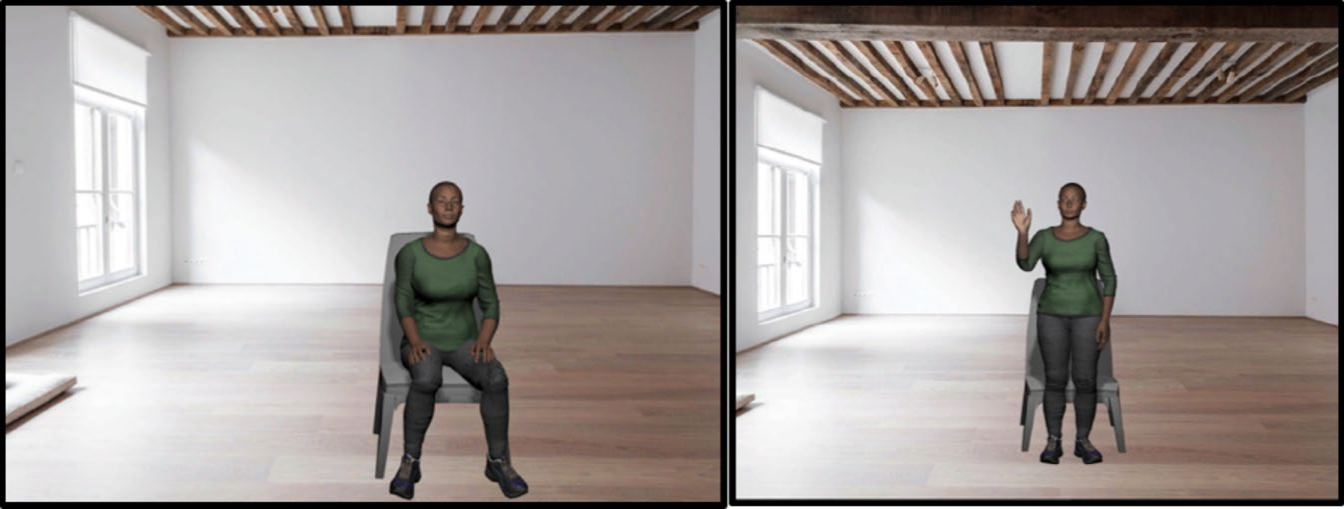
Screenshots of the virtual coach displaying multiple gestures while interacting with the participants during the exercise session.

The system development along with character control is done in the game engine - “Unity”^65^. A free source – “Mixamo” is used for animations for our coach^66^. The virtual coach is a high-quality, post-processed, realistic character with human appearance and speech (with verbal and nonverbal dialogue skills) (Fig. 1). The virtual coach displays multiple animations such as idle, standing, pointing, sitting, standing, acknowledging, nodding, looking around, shifting weight, and waving. It also allows direct manipulation of skeleton model joints (e.g., the neck joint and the spine joint). The virtual coach speaks in a natural human voice using Voice-over. This is based on previous research that suggests synthesised voice for a virtual coach is disliked by participants^18^. Dialogues are scripted and pre-recorded by a professional voice artist. The virtual coach synchronises the voice and the mouth movements. The gesticulations along with lip-syncing form the basis for a naturalistic experience for our users where they can interact with a virtual coach as they would with a human coach. The virtual environment is carefully selected for purpose of relaxation.

**Fig. 2:**
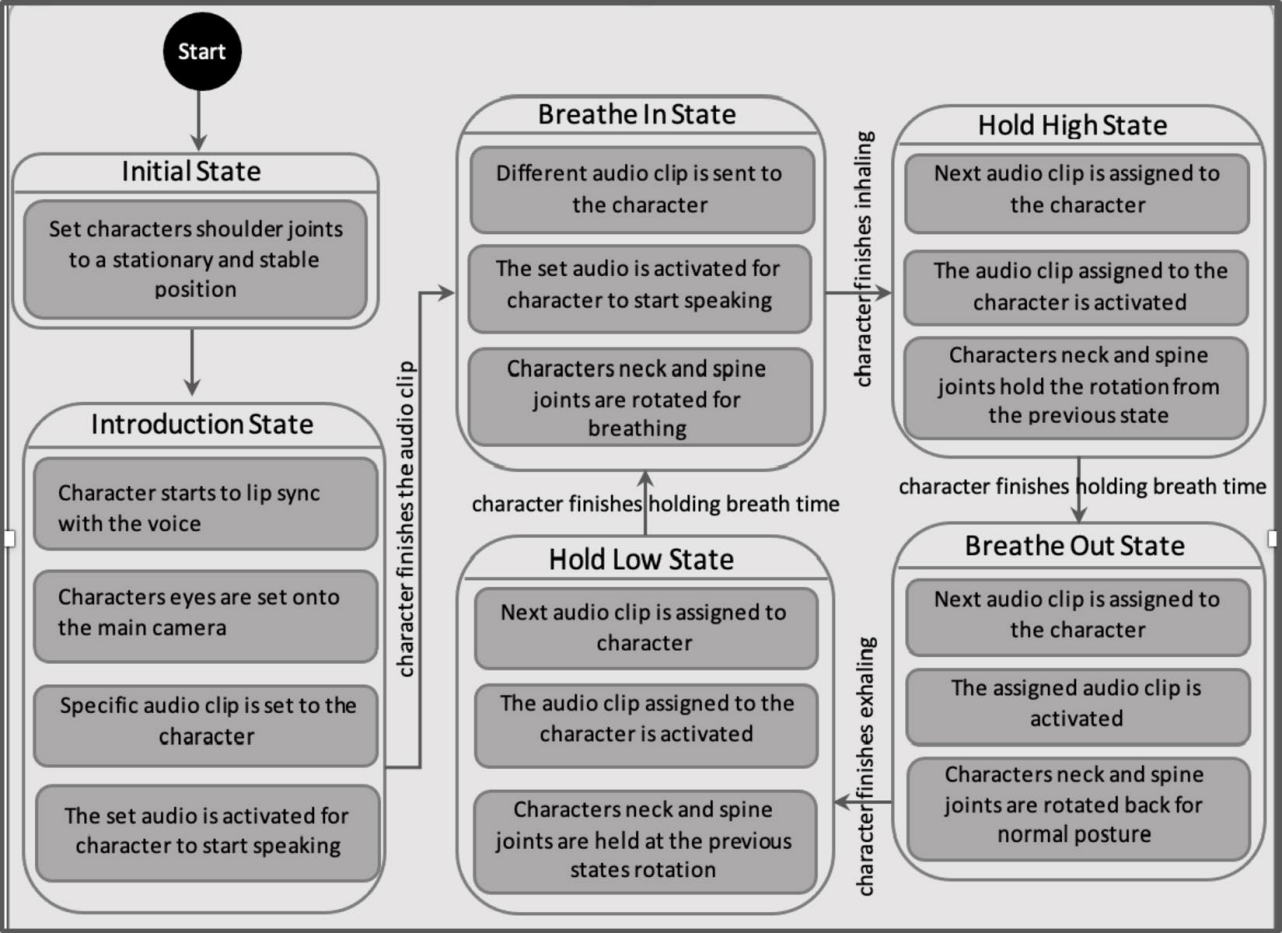
Finite state machine for controlling the breathing cycle of the virtual coach where the initial state sets the joints positioning ready for a smooth breathing animation that looks natural. The program then transitions to the introduction state where the character’s eyes are set to camera which gives a perception of characters eyes looking at the user. The character also performs lip-syncing with voice. In the inhale, hold, exhale and hold states, the neck, and spine joints are manually controlled to mimic breathing animation, and the character is constantly triggered to lip-sync with new audio in each state.

A set of finite state machines are used for the overall control of the character and the flow of the interaction^67^, such as for scene switching, flow control, character control including animations and speech, condition-based progression such as participant’s identification check, audio check, and the questionnaire data handling where the participant responses are stored to an online location. Figure 2 shows an example of one of the finite state machines used in the system that controls the breathing cycle of the virtual coach with the breathing movements, breathing instructions, and time per state.

### Design of the breathing exercises

The breathing exercises that our coach uses are based on an established breathing exercise regime known as Box Breathing^68^, where the virtual coach instructs the users on when to inhale, hold their breath, exhale, and hold their breath again. A typical breathing rate varies from 12 to 20 breaths per minute while a typical Box breathing exercise aims at 3.75 breaths per minute. Each phase in Box breathing exercise (inhale, hold, exhale, hold) is designed to last 4 seconds for controlled breathing; however, it can be challenging for some to maintain each phase for 4 seconds. Therefore, we used two variations: 3-second and 2-second phases. On one hand, the 3-second variation is not too fast to be difficult, yet long enough to slow down the breathing rate. On the other hand, the 2-second variation is slow enough for people to follow easily. While it might seem that longer cycles give more time, they demand greater concentration and control, which can be difficult for some participants. The 3-second Box breathing exercise should ideally relax participants more compared to the 2-second variation. However, the 3-second phases might still be challenging for some, as it reduces the breathing rate from a normal 12 breaths per minute to 7.5 breaths per minute.

The coach uses both verbal instructions as well as nonverbal cues including breathing physiological movements. The breathing exercises are implemented using manipulation of the neck and spine joints of the character which mimic the breathing physiology. These manipulations are timed based on the length of the inhale, hold, exhale and hold of the breathing exercise.

### User study methods

We conducted an exploratory within-subjects user study with the aims of (1) investigating the effectiveness of the virtual human as a breathing coach, (2) investigating the perception of the virtual coach, and (3) finding out the optimal cycle length of the breathing exercise. We also evaluated how well the system ran for the participants. Subjective measures using questionnaires captured participants’ personal experiences and perceptions. These measures are commonly used in human-computer interaction (HCI) studies to gauge user experience, including aspects like realism, helpfulness, and overall satisfaction. In our study, the independent variables were the length of the breathing cycle, while the dependent variables included the effectiveness of the virtual coach, participants’ perception of the virtual coach, and the optimal cycle length for the breathing exercises. For detailed questions see Sect. 5 and supplementary material.

Data were collected from 20 non-student healthy participants (10 male and 10 female). Non-student mailing lists were used for recruitment. Hence, the participants were from a pool of researchers and academic staff at the university. No demographic information such as occupation or age was collected. However, all participants were selected after a pre-screening confirmation for age (between 18 and 65 years), no breathing disorder, corrected or normal vision and hearing, and access to personal computers and the internet.

### Apparatus

Our study is based on a game development platform to implement and deploy a realistic 3D virtual coach that can be interacted with in a 2D environment on a screen or using a head-mounted display for a 3D immersion. The implementation allows the system to run standalone in the web browser using Unity’s WebGL build. Participants ran the system on their own computers without wearing a head-mounted display. They were provided with study instructions that were designed to be clear and easy to understand, minimising the risk of errors. We also used timestamping for the start and end of participation which allowed us to verify that the experiment was completed within appropriate time. We opted for non-immersive VR instead of immersive VR using head-mounted displays (HMDs) due to several practical considerations. Firstly, the necessary immersive VR gear, such as HMDs, is not readily available to all participants. This limitation would have significantly reduced our participant pool, as many potential participants do not have access to such equipment. By using non-immersive VR, we ensured that our study was more inclusive and accessible to a broader range of participants. In addition, the realistic setting better reflects real-world conditions and enhance the ecological validity of the study compared to a controlled lab environment. By allowing participants to complete the experiment in their natural environment, we aimed to capture more authentic behaviours and responses.

So far similar studies have either used a video of a virtual agent or a picture of the agent to investigate the efficiency of the virtual agent as a coach to investigate the perception of the virtual agent.

Our experiment runs in all web browsers and is hosted on GitHub. For ease of participation and to remove any chances of cross-contamination, the study was designed such that there was no physical contact between the investigator and the participant. For ease of storage and accessibility, the data was stored online.

### Experimental procedure

**Fig. 3:**
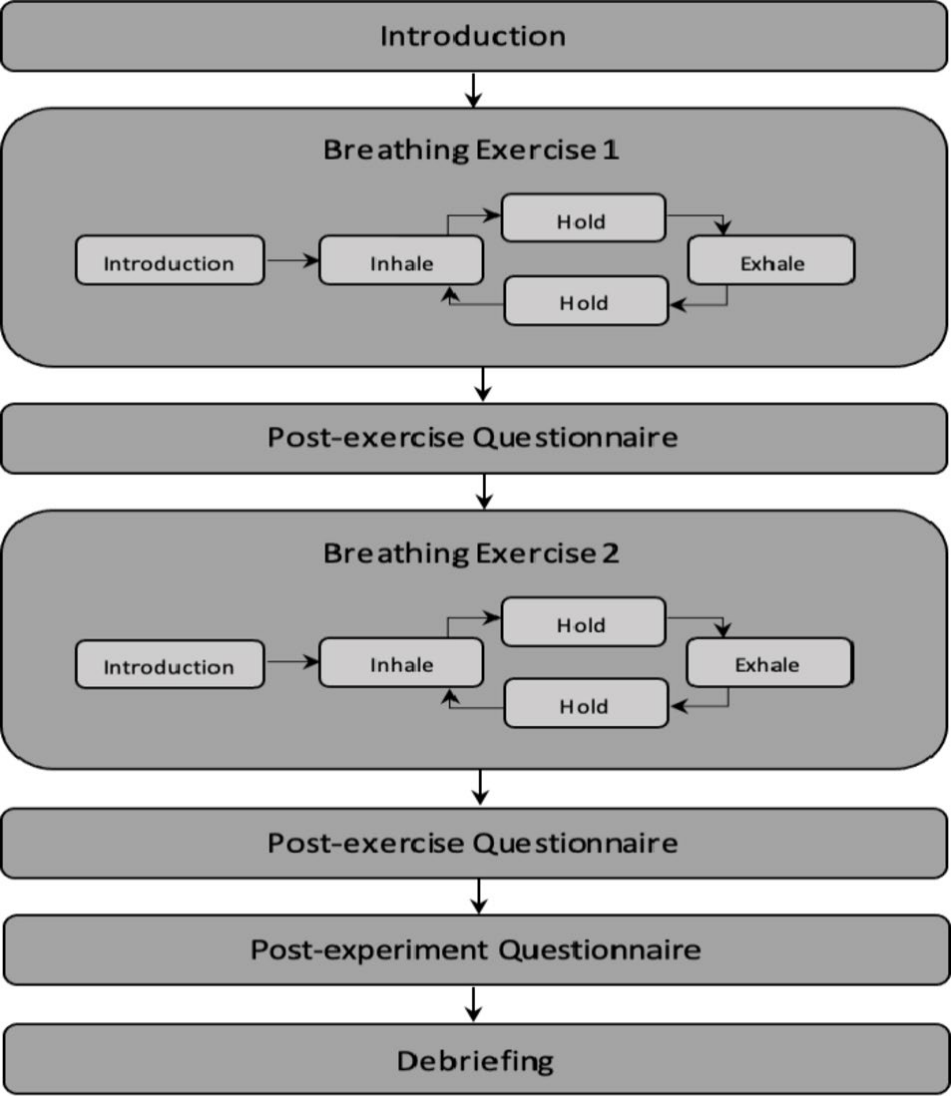
The sequence of a user’s session represented as a hierarchical finite state machine - starting with the first breathing exercise, followed by the post-exercise questionnaire, breathing Exercise 2, post-exercise, and post-experiment questionnaire, and lastly debriefing.

During the experiment, the virtual coach is introduced to the participant as the breathing instructor who will instruct and guide the participant’s breathing. The virtual coach instructs the participants using voice instructions and a variety of facial and body gestures. In the session, the virtual coach sits on a chair in an immersive room setting, looking at the participant while giving breathing instructions. All the participants were told that they can exit the experiment at any moment in time. Each participant performs two Box breathing exercises – a longer breathing exercise and a shorter breathing exercise, with each exercise lasting around 4 min. The order of the exercises was randomised between participants. The longer breathing exercise (3 + 3 + 3 + 3) has 3-seconds inhale, 3-seconds hold, 3-seconds exhale and 3-seconds hold cycle, and a shorter breathing exercise (2 + 2 + 2 + 2) has 2-seconds inhale, 2-seconds hold, 2-seconds exhale and 2-seconds hold cycle. This is based on the National Health Services recommendation of a standard breathing exercise that can be done to attain relaxation^69^. Instead of the conventional Box Breathing method, which involves 4-seconds of inhaling, holding, exhaling, and holding^70^, we simplified the process for participants by reducing it to 2 and 3 s. This adjustment is based on a standard breathing cycle, which typically consists of inhaling (1 to 1.5 s), exhaling (1.5 to 2 s), and pausing (1 to 2 s), lasting approximately 3 to 5 seconds per breath^71^. At the end of the experiment, the virtual coach thanks the participant and informs the participant about the purpose of the study before they are prompted to exit by telling them to close the window. Figure 3, shows the sequence of execution throughout the session.

At the end of each breathing exercise, participants complete a *post-exercise* questionnaire, and at the end of the experiment a *post-experiment* questionnaire. Each post-exercise questionnaire has 10 items assessing the breathing exercises, the virtual coach’s influence, and the level of relaxation. The post-experiment questionnaire has 11 items about the virtual coach’s realism and the overall experience of using the system. Participant responses were recorded using a slider that allowed continuous input. There was an open-ended question at the end of each questionnaire.

Some of the questions were adapted from validated questionnaires such as the usability questions adapted from the System Usability Scale (SUS) questionnaire^72^, while virtual coach’s influence questions were designed for the purpose of the study as there are no standardised virtual human breathing influence questionnaires.

## Results

The empirical study addresses the research questions of how well participants liked and trusted the virtual coach, how effective the virtual human was as a breathing coach, and how well the system run overall. Multiple analyses, including exploratory correlations analyses, were conducted using the software tool R^73^.

### Perception of the virtual coach

As the goal of the system was to provide an interaction experience similar to an interaction with a real human, we wanted to better understand how participants perceived the virtual coach. This included qualities typically associated with human rapport, as well as the realism of the virtual coach. Therefore, we investigated how much participants liked and trusted the coach. We also investigated how realistic they found multiple aspects of the coach which are detailed below.

### Likeability and trust

**Fig. 4:**
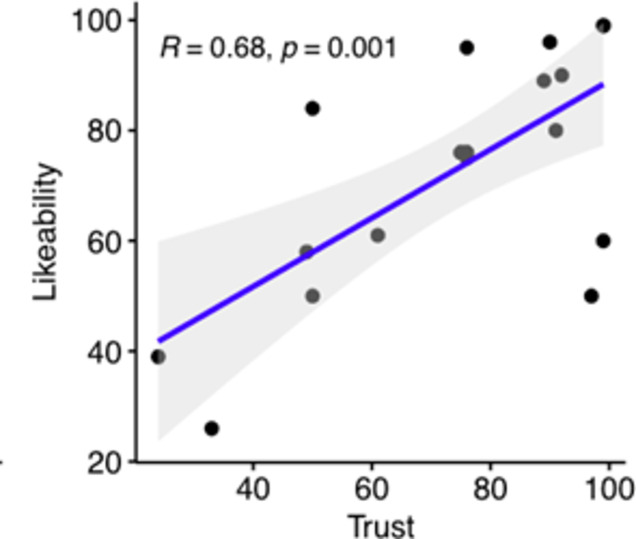
We found a strong positive correlation between trusting and liking the coach.

Likeability and trust are important qualities of a coach and therefore need to be considered in the implementation of virtual humans. We investigated whether participants liked (“How would you rate the coach?“: “Very Likeable” -- “Not Likeable at all”) and trusted (“How would you rate the coach?“: “Very Trustworthy” -- “Not Trustworthy at all”) the virtual coach. The average likability score was 72.75, while trustworthiness scored 73.85. These scores are measured on a scale from 0 to 100. While these scores are not directly comparable to the System Usability Scale (SUS), which considers scores above 68 as above average, they do suggest a generally positive perception of the coach. Specifically, the likability score of 72.75 and the trustworthiness score of 73.85 indicate that participants had a favourable view of the coach. This interpretation is supported by the fact that these scores are above the midpoint of the scale, suggesting a positive response overall. The two measures further showed a strong positive correlation with *R* = 0.68 and *p* = 0.001 (Fig. 4).

### Realism

**Fig. 5:**
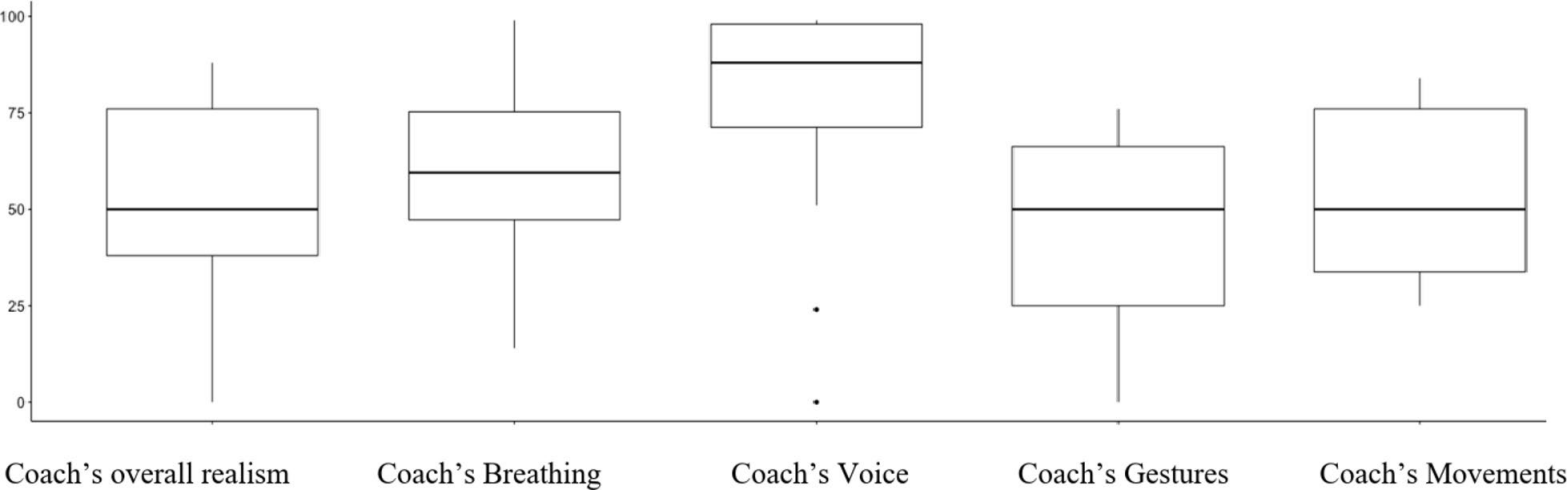
Overall realism score for the Virtual Coach and the scores for the different facets of realism.

We assessd how realistic the participants found the virtual coach overall, as well as how realistic the four key aspects of: breathing motion, gestures, movements, and voice were perceived (Fig. 5). We asked participants five questions on *“How Realistic did you find: 1) The Coach*,* 2) Breathing*,* 3) Gestures*,* 4) Voice and 5) Movements on a scale of Very Realistic to Not Realistic at all”.* We analysed the responses on a scale from 0 to 100, where 0 means least realistic and 100 means most realistic. The highest mean score was for coach’s voice (77.25), while for overall realism the mean was 53.8.

**Fig. 6:**
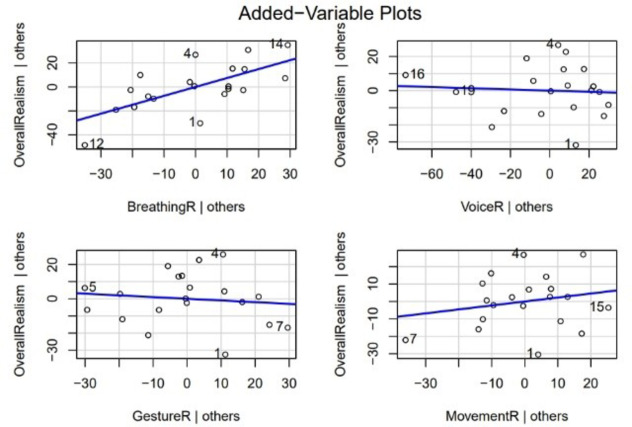
Added-variable plots showing the partial relationship between each realism component (breathing, voice, gesture, and movement) and overall realism, holding the other predictors constant.

To examine how different aspects of realism -- breathing, voice, gesture, and movement -- predict the overall realism scores, we ran multiple linear regression analysis. The multiple linear regression model was statistically significant (F(4,15) = 13.36, *p* < 0.001) and explained approximately 72% of the variance in overall realism scores (adjusted R^2^ = 0.72). The key findings are that the breathing realism had a significant positive effect on overall realism (β = 0.74, *p* = 0.003), indicating that higher breathing realism was associated with higher overall realism, while as the voice realism (β = −0.03, *p* = 0.814), gesture realism (β = −0.10, *p* = 0.674), and movement realism (β = 0.23, *p* = 0.410) did not have statistically significant effects on overall realism. The added-variable plots (Fig. 6) provide a visual representation of these relationships, showing the association between each predictor and the outcome variable while controlling for the other predictors.

Before examining the predictive power of our model, assessment of its foundational assumptions was undertaken to affirm the integrity of our analysis. Diagnostic tests including residual analysis and variance inflation factors (VIF) confirmed the appropriateness of the model, with all VIF values below 2.12 indicating acceptable levels of multicollinearity. The normality assumption was met as evidenced by the Q-Q plot of residuals, supporting the validity of the statistical inferences.

These findings suggest that breathing realism plays a crucial role in overall perceived realism, which has important implications for the development of realistic virtual humans.

### The role of realism

**Fig. 7:**
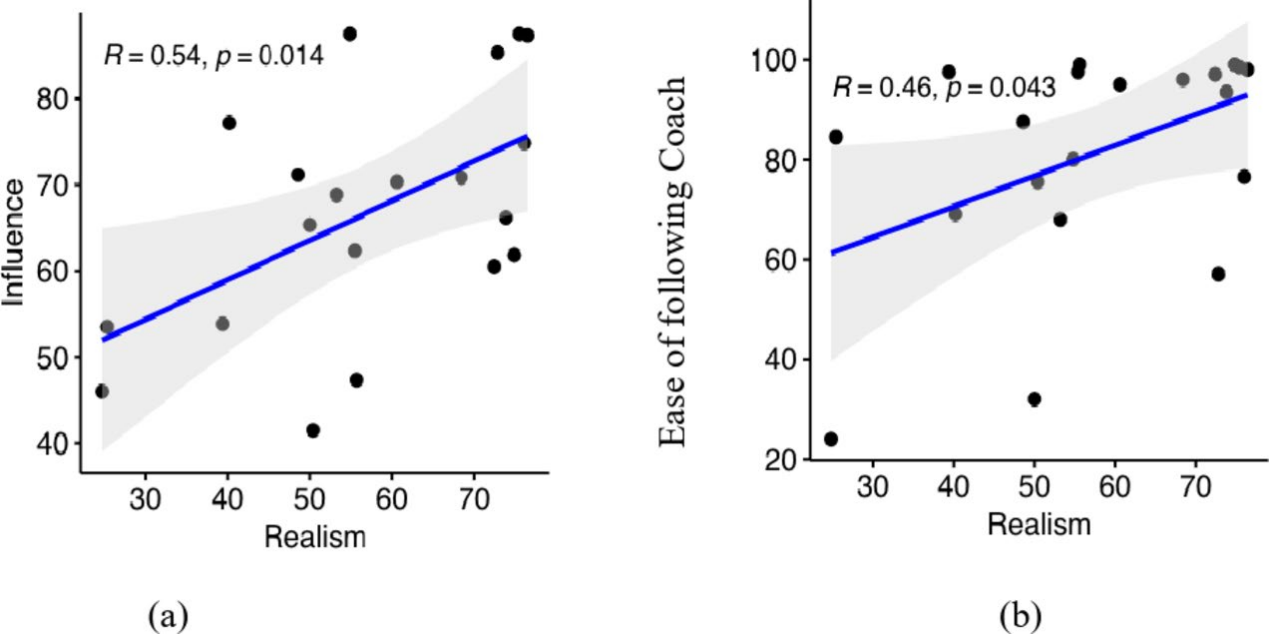
Relationship between Realism of the character on the one hand and the Influence and Ease of following Coach on the other hand.

In many virtual human based systems, a high level of realism is seen as desirable in and by itself^74,75^. In our system, the virtual human has the clear function of guiding the participant through breathing exercises. Given this functional role, we are interested in understanding the importance of the realism of the character. We found a significant, positive correlation between realism and both the influence felt by the participant from the virtual coach with *R* = 0.54 and *p* = 0.014 (Fig. 7a ) and the ease of following the coach with *R* = 0.46 and *p* = 0.043 (Fig. 7b).

This relationship suggests that the efficiency of our coach — as defined by the influence felt by the participants — and the ease with which they followed the coach improved as the perceived level of realism increased.

### Effectiveness of the virtual coach for exercise training

As a breathing coach, the virtual human should be able to influence the user such that they persistently follow its guidance. Since the coach uses both verbal and nonverbal cues, we wanted to look at the strength of the influence between verbal and nonverbal influence. We asked participants to score the influence felt from the coach, the ease with which they followed the coach and the level of relaxation.

The aim is to compare the effect of a longer breathing cycle and a shorter breathing cycle on getting influenced by the coach.

Our hypotheses are that a longer breathing cycle will firstly, lead to a stronger influence from the coach, secondly, be difficult to follow the coach and thirdly, achieve a higher level of relaxation compared to shorter breathing cycle. This is because longer breathing cycle requires more focus, endurance and breath control, and therefore can be harder to sustain especially for beginners, making participants more reliant on the coach’s guidance to maintain the rhythm and technique.

**Fig. 8:**
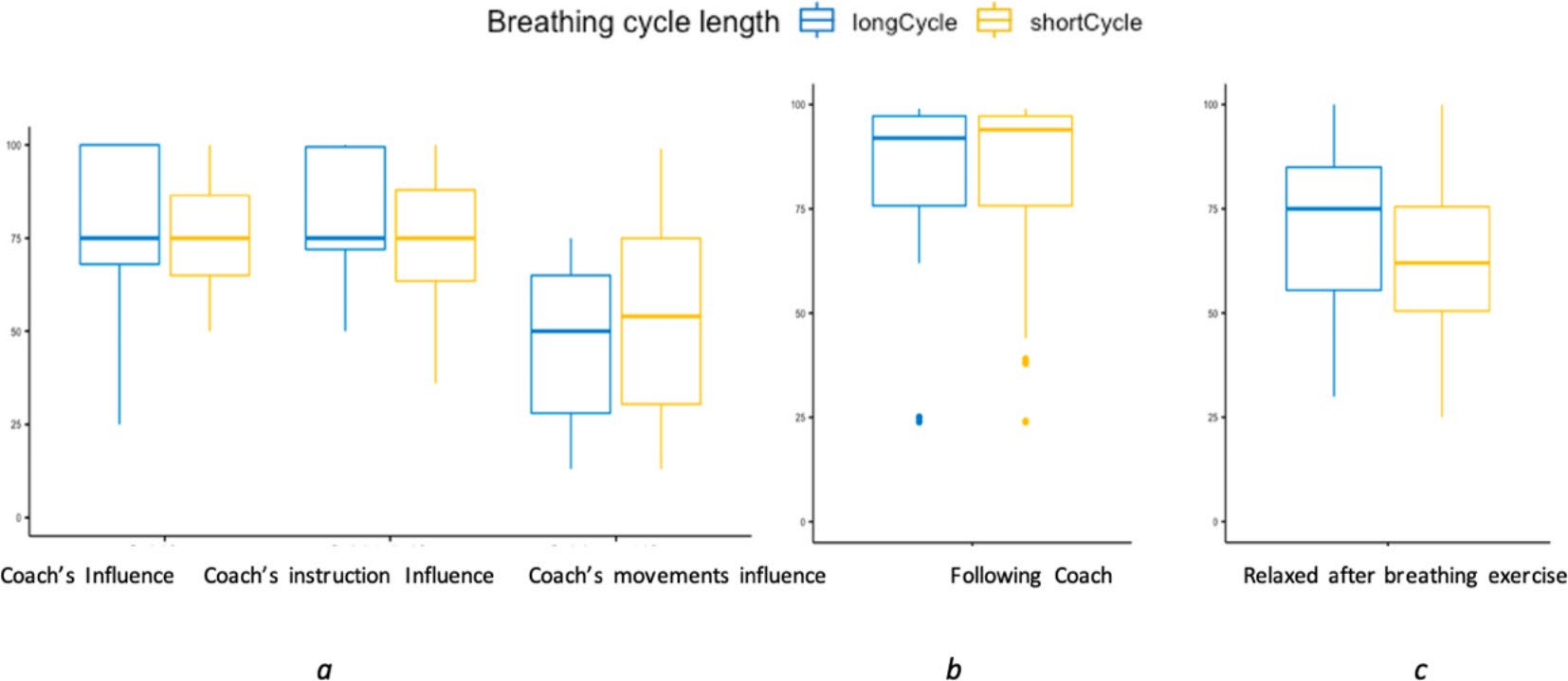
(**a**) Coach’s Influence, (**b**) Ease of following the Coach and (**c**) Level of relaxation reported by participants between the exercises.

A paired sample t-test revealed no significant difference between the short cycle and long cycle for the coach’s overall influence. We investigated the influence felt by the participant by looking into how much the coach’s instructions and the coach’s breathing movements influenced the participant. Similar to overall influence the paired sample t-test revealed no significant difference between the short cycle and long cycle for the coach’s Instructions Influence, and the Coach’s breathing movements influence (Fig. 8a).

No significant difference in the coach’s influence and in ease of following the coach was found between the short and long cycle exercises. Similarly, no significant difference was found in the level of relaxation between the two exercises (M = 7.8, t(19) = 0.93, p-value = 0.36). However, the overall easiness of following the coach (Fig. 8b) had a mean of 81.25 and level of relaxation had a mean of 66.25 (Fig. 8c).

The virtual coach was rated as easy to follow and capable of inducing relaxation in the participants. This begs the question if participants are willing to replace a human coach with a virtual coach. To analyse this, we split the responses into three groups - “Not very likely” (0–32), “not sure” (33–66), and “Very Likely” (67–100). 8 participants answered, “Very Likely”, 8 participants answered, “Not Sure”, and 4 participants answered, “Not very likely”. The result of a chi square test, however, was not significant (X^2^ (2) = 1.6, p = .44).

### The breathing exercises

**Fig. 9:**
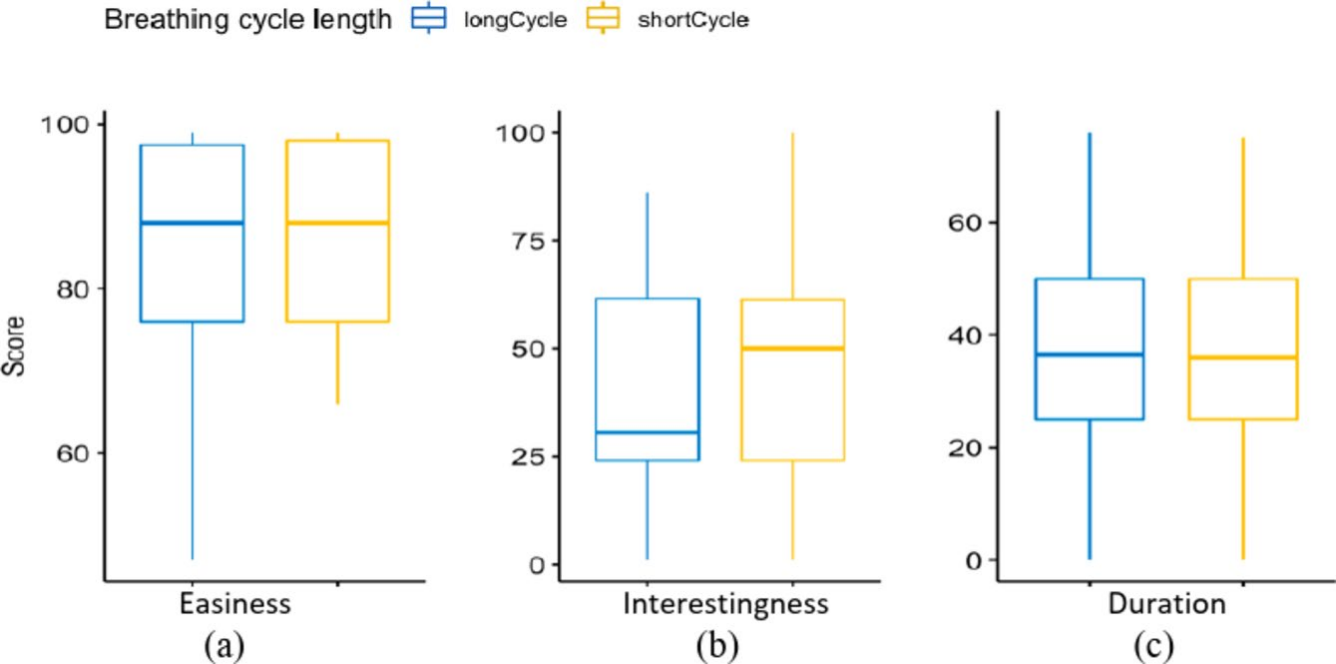
A two- sample t-test reveals a significant difference in (**a**) easiness, (**b**) interestingness, and (**c**) duration of the breathing exercise, however no significant difference between long and short exercises.

A key part of the empirical study was investigating the optimal length of the breathing cycle as it will vary between participants, but little information on this is available from prior studies. We compared two different cycle lengths of the breathing exercises to understand the effect on the level of easiness, interestingness, and level of relaxation (Fig. 9) by asking them “How did you find the breathing exercise?”

Our hypotheses are that longer breathing exercise cycles will be firstly, difficult to perform since it involves inhaling, holding the breath and exhaling for a longer duration and secondly, more interesting compared to short bursts of monotonous inhalation and exhalation. We also hypothesize that, the exercise with longer cycles will feel overall shorter than short cycles.

A two-sample t-test revealed no significant difference between the long cycle and the short cycle breathing exercise neither for easiness (*p* = 0.51), interestingness (*p* = 0.69), nor duration (*p* = 0.84).

Irrespective of the difference between the two exercises, we wanted to investigate how the interestingness, easiness, and duration of the breathing exercises correlate to the level of relaxation that was felt by the participants.

**Fig. 10:**
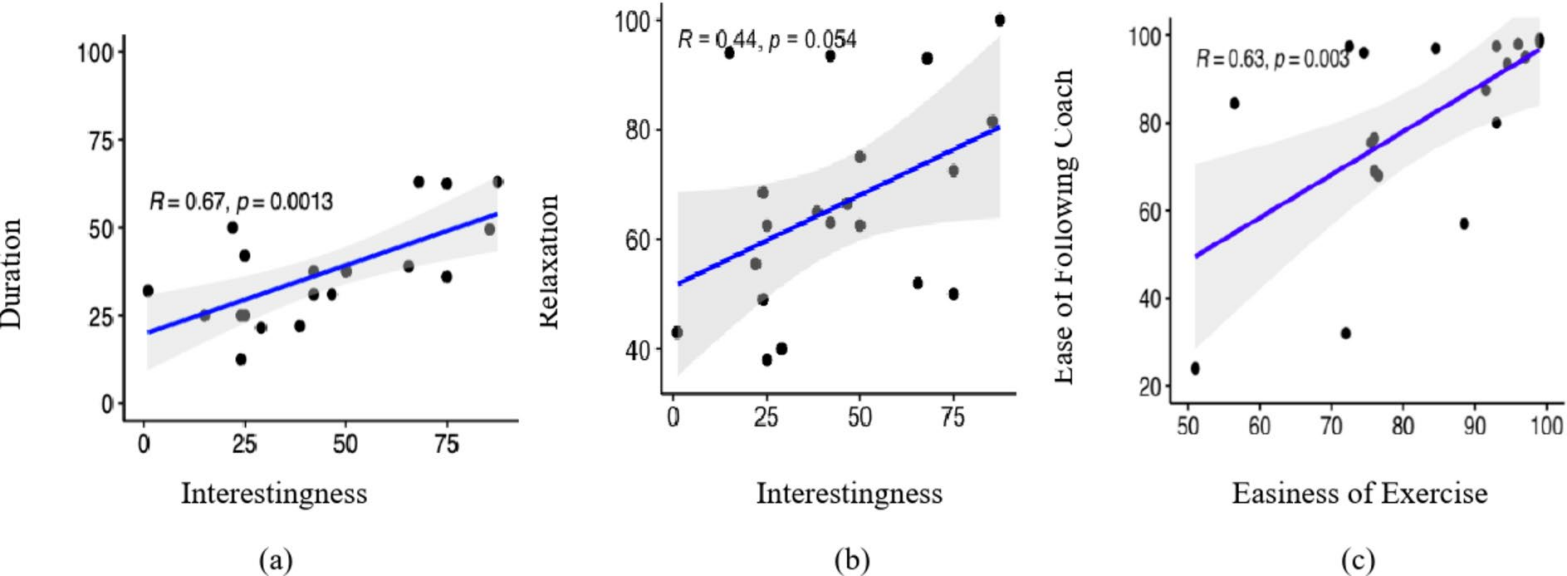
Relationship between (**a**) Interestingness of the exercise and duration of breathing exercise), and (**b**) level of Relaxation, respectively. (**c**) Perceived easiness of the exercise vs. ease of following the coach.

Looking at both exercises combined, we found the expected effect that the interestingness of the breathing exercises strongly and significantly correlated with how appropriate participants rated the duration of an exercise (*R* = 0.67 and *p* = 0.001) (Fig. 10a). More importantly, we found a significant positive correlation with the level of reported relaxation (*R* = 0.44 and *p* = 0.05) (Fig. 10b). Lastly, the participants that found the exercise easy to do, also found it easy to follow the coach (*R* = 0.63, *p* = 0.003) (Fig. 10c).

### Usability of the system

The participants interacted with the virtual coach in real-time via a computer screen on their computer. It is hence important to assess how well the system ran for participants. We asked participants -- “*How well did the system run?”: “*Not Well at all” to “Very Well”. The responses were scored from 0 to 100, where 0 means Not at all and 100 means Very Much. The mean score for usability of the system was 76.15 with a standard deviation of 18.45. To determine potential future use, we asked participants -- *“How much would you be interested in continuing using the system”*: “Not at all” to “Very Much”. Responses were analysed on a scale of 0–100 (with 0 being Not at all and 100 being Very much). The mean score was 60.4 and standard deviation was 22.65. In order to investigate this further, we binarized the responses and ran a chi square test. The analysis showed a significant bias towards a yes on intention to use the system again (X^2^ (1) = 5, *p* = 0.02).

## Discussion

We developed an online real-time relaxation system that uses an established breathing exercise led by a coach, which is a virtual human delivering verbal instructions and nonverbal cues. The system uses the concepts of breathing exercises for relaxation as well as scientific evidence corroborating the advantages of virtual coaches in health and wellbeing. By displaying breathing movements on our virtual coach, we aim to harness the mechanism of physiological entrainment, making maximal use of embodied cues that a virtual human can provide^51^. An evaluation study with healthy adults investigated the perception of the virtual human coach, the breathing exercise as well as the overall usability of the system.

### The virtual coach

While the overall realism was not rated as maximally high, the coach was liked and trusted by the participants, implying that a very high level of realism of a virtual human is not a prerequisite for it to be liked and trusted. However, this does not mean that realism is not an important aspect of virtual human-based systems, since we found that the level of realism affected how much participants felt influenced by the coach and how easy they found it to follow the coach (Fig. 7). A recent study suggests that a higher degree of realism of individual facets is preferred regardless of whether this leads to a realism mismatch between the facets^59^. We analysed multiple facets of realism — voice, gestures, and movements — and found the coach’s voice is perceived as the most realistic aspect. Given that the voice was an actual human recording, we see this as a manipulation check and as establishing a baseline against which virtual attributes can be compared.

While participants reported that they felt most influenced by the verbal instructions, they still reported a relatively large influence from the coach’s breathing movements (Fig. 8a). Given that these are more subtle signals this was an important finding since it corroborates our assumption that realistic embodiment is important for effective training. A higher level of immersion, e.g., through the use of head-mounted displays as well as more naturalistic movements might additionally increase the influence these signals play. The latter assumption is supported by our exploratory finding that perceived realism correlates with influence. It is worth noting that, while likeability and trust showed a strong positive correlation (Fig. 4), none of them individually correlated to the level of relaxation or to the ease of following the coach.

### The exercises

At the core of our system itself are the “Box” breathing exercises that participants perform. As there is very little systematic data available on what breathing cycle length and exercise lengths are appropriate for healthy participants to increase the level of relaxation, it was vital for us to investigate how participants perceived the impact of the length of the breathing exercise. To establish a baseline and as a segue for later system iteration with adaptive exercises, we compared two lengths of breathing cycles. Although we varied the lengths of the breathing cycles in the exercises, both exercises were rated as being on the short side (Fig. 9c). We found no significant difference between the two exercises in any of the measures i.e., interestingness, duration, easiness (Fig. 9), influence from coach, and ease of following the coach (Fig. 8) which indicates that the difference in cycle length between the two exercises might have been too small. On the upside, we found a (non-significant) trend that the exercises with longer breathing cycles lead to a higher level of relaxation. This is in line with findings that longer and deeper breathing is more suitable for relaxation purposes^42^. Irrespective of the exercise cycle lengths, our exploratory analysis found that the rating of the appropriateness of the duration of the breathing exercise had a strong positive correlation with the interestingness, meaning that the more appropriate participants found the duration of the exercise, the more interesting they found it (Fig. 10).

### Usability of VR-based breathing training system

All participants ran our real-time system on their personal computers without special VR equipment or any supervision. In such configuration usability and acceptability are of key importance since the working of the system depends on users’ hardware and the novelty of the technology. The fact that 20 participants completed the experiment is evidence that our system is technically and conceptually fit for the purpose. Our goal is for the breathing coach to bridge the gap between healthcare interventions and technological developments. The use of virtual reality and virtual humans provides unique new opportunities, and we see our system as an important step towards improving access to physical as well as mental health help in a broader population not just limited to relaxation. It was very encouraging to see that our users would the interested in continuing to use the system, and two-thirds of them would accept a virtual coach in place of a real human coach. The high level of usability combined with trust, likeability and influence from the coach lends support for the research carried out in this study

### Limitations and future work

The low immersiveness of the desktop version of our current system will limit the level of immersion and, consequently, the level of influence and level of relaxation that can be achieved. As VR equipment is becoming more widely available to end users, it will be increasingly more practical to run 3D immersive studies. Therefore, we aim to investigate the virtual breathing coach using 3D immersive VR technology in future studies. Another limitation of our current system is that the relaxation level is solely measured through users’ explicit assessment. To address this issue, we plan to use physiology-based measures of relaxation such as breathing rate and galvanic skin response.

A key feature of our system is that it is based on real-time control and rendering. While in the presented form the system is feed-forward only, the real-time capabilities lay the foundation for future closed-loop systems that consider explicit and implicit user feedback.

Explicit user input will allow adapting the difficulty level of the exercise to maximise training efficiency at the individual level. Implicit input based on the users’ physiology can directly measure the influence the coach has on the breathing behaviour and adapt the exercises accordingly. It will also allow tailoring the exercise regime such as to maximise the user’s level of relaxation. These inputs, in turn, will allow harnessing the mechanism of physiological entrainment, making maximal use of embodied cues that a virtual human can provide^76^.

Our study evaluates the system’s usability as a tool for breathing relaxation exercises guided by a virtual coach. While relaxation exercises can be performed on demand, medical conditions like asthma require specific usage guidelines, necessitating professional guidance. Therefore, we plan to collaborate with medical experts to assess the system’s effectiveness as a medicinal practice tool.

## Conclusion

In summary, extensive research in psychology, human-computer interaction, human virtual human interaction, and related fields affirms the value of realistic 3D avatars. These avatars boost engagement, communication, empathy, and specific application outcomes across various domains. Realistic virtual humans, resembling human features, are crucial for high-fidelity simulations, especially in fields like healthcare^77^.

Our research demonstrated that even virtual agents with limited intelligence and realism can effectively serve as coaches for physical exercise training. Our virtual coach proved to be an effective tool for influencing participants’ breathing patterns and was well-received, with participants expressing a willingness to use it again. The virtual coach was trusted and liked, enhancing its potential as a reliable breathing intervention. However, variations in the breathing exercise cycle lengths did not yield significant differences, likely due to the minimal differences in cycle lengths. Overall, the virtual coach shows promise for future applications in improving physical and mental health through guided breathing exercises. Looking ahead, as virtual humans become increasingly photorealistic, it presents both challenges and substantial potential. Challenges include maintaining consistency in appearance and behaviour, and the ethical considerations in their design and use, while the potential lies in achieving human-machine interaction that rivals human-to-human interaction in terms of seamlessness, and naturalness.

## Electronic supplementary material

Below is the link to the electronic supplementary material.

Supplementary Material 1

## Data Availability

The datasets generated during and/or analysed during the current study are available from the corresponding author on reasonable request.

## References

[1] *Long-term effects of coronavirus (long COVID). NHS. *Available at: https://www.nhs.uk/conditions/covid-19/long-term-effects-of-covid-19-long-covid/ (accessed Dec. 13, 2024).

[2] Whelan, C. (2021) *The best breathing exercises for COVID-19,**Healthline. *Available at: https://www.healthline.com/health/breathing-exercises-for-covid (accessed: Dec. 13, 2024).

[3] Ley, R. The modification of Breathing Behavior. *Behav. Modif.* **23** (3), 441–479 (1999). 10467892 10.1177/0145445599233006

[4] Sankar, J. & Das, R. R. Asthma – A Disease of How We Breathe: Role of Breathing Exercises and Pranayam, *Indian Journal of Pediatrics*, vol. 85, no. 10. Springer, pp. 905–910, doi: (2018). 10.1007/s12098-017-2519-6 10.1007/s12098-017-2519-6 29247426

[5] Brown, R. P. & Gerbarg, P. L. Yoga breathing, meditation, and longevity. *Ann. N Y Acad. Sci.* **1172** (1), 54–62. 10.1111/j.1749-6632.2009.04394.x (Jul. 2009). 10.1111/j.1749-6632.2009.04394.x 19735239

[6] van Coller-Peter, S. & Manzini, L. Strategies to establish rapport during online management coaching. *SA J. Hum. Resour. Manag*. **18**, 1–9. 10.4102/sajhrm.v18i0.1298 (2020).

[7] Singhai, K., Swami, M. K., Nebhinani, N., Rastogi, A. & Jude, E. Psychological adaptive difficulties and their management during COVID-19 pandemic in people with diabetes mellitus. *Diabetes Metab. Syndr. Clin. Res. Rev.* **14** (6), 1603–1605. 10.1016/j.dsx.2020.08.025 (2020). 10.1016/j.dsx.2020.08.025 PMC7443210 32862099

[8] Sui, W., Rush, J. and Rhodes, R.E. (2022) ‘Engagement with web-based fitness videos on YouTube and Instagram during the COVID-19 pandemic: Longitudinal Study’, JMIR Formative Research, 6(3). 10.2196/25055. 10.2196/25055 PMC8906834 35258459

[9] Gavish, B. Device-guided breathing in the home setting: Technology, performance and clinical outcomes, *Biol. Psychol.*, vol. 84, no. 1, pp. 150–156, Jul. doi: (2010). 10.1016/j.biopsycho.2010.02.013 10.1016/j.biopsycho.2010.02.013 20193729

[10] Catalyst, N. What is Telehealth? *Innov. Care Deliv*, (2018).

[11] Yue, J. L. et al. Mental health services for infectious disease outbreaks including COVID-19: A rapid systematic review, *Psychological Medicine*, vol. 50, no. 15. Cambridge University Press, pp. 2498–2513, doi: (2020). 10.1017/S0033291720003888 10.1017/S0033291720003888 PMC7642960 33148347

[12] Bickmore, T. W., Puskar, K., Schlenk, E. A., Pfeifer, L. M. & Sereika, S. M. Maintaining reality: relational agents for antipsychotic medication adherence. *Interact. Comput.* **22** (4), 276–288. 10.1016/j.intcom.2010.02.001 (2010).

[13] Morilla, M. D. R., Sans, M., Casasa, A. & Giménez, N. Implementing technology in healthcare: insights from physicians. *BMC Med. Inf. Decis. Mak.* **17** (1). 10.1186/s12911-017-0489-2 (2017). 10.1186/s12911-017-0489-2 PMC5488364 28655299

[14] Friebe, M. & Otto-von-Guericke-Universität Magdeburg, R-P-Ö. International healthcare vision 2037 new technologies, educational goals and entrepreneurial challanges: proceedings + summary of the 5th BME-IDEA EU Conference, 11–13 June 2017, Magdeburg, Germany, no. June, (2017).

[15] Blandford, A. HCI for health and wellbeing: challenges and opportunities. *Int. J. Hum. Comput. Stud.* **131**, 41–51. 10.1016/j.ijhcs.2019.06.007 (2019).

[16] Latif, S., Qadir, J., Qayyum, A., Usama, M. & Younis, S. Speech Technology for Healthcare opportunities challenges and State of the Art.pdf. *Biomed. Eng. (NY)*, **14**, (2021). 10.1109/RBME.2020.3006860 32746367

[17] Hawley, L. L., Rector, N. A., DaSilva, A., Laposa, J. M. & Richter, M. A. Technology supported mindfulness for obsessive compulsive disorder: self-reported mindfulness and EEG correlates of mind wandering. *Behav. Res. Ther.* **136**10.1016/j.brat.2020.103757 (2021). 10.1016/j.brat.2020.103757 33310604

[18] Shamekhi, A. & Bickmore, T. Breathe deep: A breath-sensitive interactive meditation coach, in *ACM International Conference Proceeding Series*, pp. 108–117, doi: (2018). 10.1145/3240925.3240940

[19] Shamekhi, A. & Bickmore, T. Breathe with Me: A Virtual Meditation Coach. In: Brinkman, WP., Broekens, J., Heylen, D. (eds) *Intelligent Virtual Agents. IVA.* **9238**. 10.1007/978-3-319-21996-7_29 (2015).

[20] Li, Q., Cao, H., Li, Y. & Lu, Y. How do you breathe-a non-contact monitoring method using depth data. *IEEE e-Health Netw. Appl. Serv.* 1–6. 10.1109/HealthCom.2017.8210796 (Jul. 2017).

[21] Brinkman JE, Toro F, Sharma S. Physiology, Respiratory Drive. In: *StatPearls.* Treasure Island (FL): StatPearls Publishing; June 5, 2023. 29494021

[22] Schleifer, L. M., Ley, R. & Spalding, T. W. A hyperventilation theory of job stress and musculoskeletal disorders, *Am. J. Ind. Med.*, vol. 41, no. 5, pp. 420–432, Jul. doi: (2002). 10.1002/ajim.10061 10.1002/ajim.10061 12071494

[23] Zaccaro, A. et al. How Breath-Control can change your life: a systematic review on psycho-physiological correlates of slow breathing. *Front. Hum. Neurosci.* **12**10.3389/fnhum.2018.00353 (2018). 10.3389/fnhum.2018.00353 PMC6137615 30245619

[24] Khalsa, S. B. S. Treatment of chronic insomnia with yoga: a preliminary study with Sleep?Wake diaries. *Appl. Psychophysiol. Biofeedback*. **29** (4), 269–278. 10.1007/s10484-004-0387-0 (Jul. 2004). 10.1007/s10484-004-0387-0 15707256

[25] Epe, J., Stark, R. & Ott, U. Different effects of four yogic breathing techniques on mindfulness, stress, and well-being. *OBM Integr. Complement. Med.* **06** (03), 1–1. 10.21926/obm.icm.2103031 (2021).

[26] Papworth Breathing (May 2018) Papworth Breathing -- Respiratory Treatment - Treatments - Physio.co.uk. Available at: https://www.physio.co.uk/treatments/respiratory-treatment/papworth-breathing.php#:~:text=The%20Papworth%20breathing%20technique%20consists%20of%20a%20series,deeply%20or%20too%20fast%20by%20emphasising%20nose%20breathing. (accessed: Dec. 13 2024)

[27] Holloway, E. A. & West, R. J. Integrated breathing and relaxation training (the Papworth method) for adults with asthma in primary care: a randomised controlled trial, *Thorax*, vol. 62, no. 12, pp. 1039–1042, Jul. doi: (2007). 10.1136/thx.2006.076430 10.1136/thx.2006.076430 PMC2094294 17573445

[28] Hwu, M. Using Box Breathing to handle tilt/stress from Gaming. *Healthy Environ.*,. (2021). Available at: https://1-hp.org/blog/hpforgamers/using-box-breathing-to-handle-tilt-stress-from-gaming/ (accessed on Dec 13. 2024)

[29] Ghandeharioun, A. & Picard, R. BrightBeat: Effortlessly influencing breathing for cultivating calmness and focus, *Conf. Hum. Factors Comput. Syst. - Proc.*, vol. Part F1276, pp. 1624–1631, doi: (2017). 10.1145/3027063.3053164

[30] Paraedes, E. P. et al. Just breathe: In-Car interventions for guided slow breathing. *Proc. ACM Interact. Mob. Wearable Ubiquitous Technol.* **2** (1), p28 (2018).

[31] Patibanda, R., Floyd, F., Mueller, M., Leskovsek & Duckworth, J. Life Tree: Understanding the Design of Breathing Exercise Games. *Life Tree*, 19–31, 10.1145/3116595.3116621 (2017).

[32] Van Rooij, M., Lobel, A., Harris, O., Smit, N. & Granic, I. DEEP: A biofeedback virtual reality game for children at-risk for anxiety, *Conf. Hum. Factors Comput. Syst. - Proc.*, vol. 07-12-May-, no. May, pp. 1989–1997, doi: (2016). 10.1145/2851581.2892452

[33] Valmaggia, L. R., Latif, L., Kempton, M. J. & Rus-Calafell, M. Virtual reality in the psychological treatment for mental health problems: an systematic review of recent evidence. *Psychiatry Res.* **236**, 189–195. 10.1016/j.psychres.2016.01.015 (2016). 26795129 10.1016/j.psychres.2016.01.015

[34] Faria, A. L. et al. Combined Cognitive-Motor Rehabilitation in virtual reality improves Motor outcomes in Chronic Stroke - A Pilot Study. *Front. Psychol.* **9**, 854. 10.3389/fpsyg.2018.00854 (2018). 29899719 10.3389/fpsyg.2018.00854 PMC5988851

[35] Krijn, M., Emmelkamp, P. M. G., Olafsson, R. P. & Biemond, R. Virtual reality exposure therapy of anxiety disorders: A review, *Clin. Psychol. Rev.*, vol. 24, no. 3, pp. 259–281, Jul. doi: (2004). 10.1016/j.cpr.2004.04.001 10.1016/j.cpr.2004.04.001 15245832

[36] Beck, J. G., Palyo, S. A., Winer, E. H., Schwagler, B. E. & Ang, E. J. Virtual Reality Exposure Therapy for PTSD Symptoms After a Road Accident: An Uncontrolled Case Series, *Behav. Ther.*, vol. 38, no. 1, pp. 39–48, Jul. doi: (2007). 10.1016/j.beth.2006.04.001 10.1016/j.beth.2006.04.001 17292693

[37] Caravas, P., Kritikos, J., Alevizopoulos, G. & Koutsouris, D. Participant Modeling: The Use of a Guided Master in the Modern World of Virtual Reality Exposure Therapy Targeting Fear of Heights, in *Lecture Notes of the Institute for Computer Sciences, Social-Informatics and Telecommunications Engineering, LNICST*, vol. 376 LNICST, pp. 161–174, doi: (2021). 10.1007/978-3-030-76066-3_13

[38] Cano Porras, D., Siemonsma, P., Inzelberg, R., Zeilig, G. & Plotnik, M. Advantages of virtual reality in the rehabilitation of balance and gait, *Neurology*, vol. 90, no. 22, p. 1017 LP – 1025, May doi: (2018). 10.1212/WNL.0000000000005603 10.1212/WNL.0000000000005603 29720544

[39] Freeman, D. et al. Mar., Virtual reality in the assessment, understanding, and treatment of mental health disorders, *Psychol. Med.*, vol. 47, no. 14, pp. 2393–2400, doi: (2017). 10.1017/s003329171700040x 10.1017/S003329171700040X PMC5964457 28325167

[40] Cikajlo, I. & Matjacic, Z. Advantages of virtual reality technology in rehabilitation of people with neuromuscular disorders. in *Recent. Adv. Biomedical Eng.*, (2009).

[41] Mcgloin, R., Farrar, K. M. & Fishlock, J. Triple whammy! Violent games and violent controllers: investigating the use of realistic gun controllers on perceptions of realism, immersion, and outcome aggression. *J. Commun.* **65** (2), 280–299. 10.1111/jcom.12148 (2015).

[42] Noble, D. J. & Hochman, S. Hypothesis: pulmonary afferent activity patterns during slow, deep breathing contribute to the neural induction of physiological relaxation. *Front. Physiol.* **10**10.3389/fphys.2019.01176 (2019). 10.3389/fphys.2019.01176 PMC6753868 31572221

[43] Matheus, K., Vazquez, M. & Scassellati, B. A Social Robot for Anxiety Reduction via Deep Breathing, *RO-MAN 2022–31st IEEE Int. Conf. Robot Hum. Interact. Commun. Soc. Asoc. Antisocial Robot.*, pp. 89–94, doi: (2022). 10.1109/RO-MAN53752.2022.9900638

[44] Wang, C. (2024) Calm robot, UBC IDEA lab. Available at: https://idea.nursing.ubc.ca/2023/01/14/stress-relief-robot/ (accessed: Dec. 13 2024).

[45] Ab Aziz, A., Fahad, A. S. & Ahmad, F. CAKNA: A Personalized Robot-Based Platform for Anxiety States Therapy, *Intell. Environ.* vol. 22, no. 13th International Conference on Intelligent Environments (IE), pp. 141–150, 2017. (2017).

[46] Saberi, M., DiPaola, S. & Bernardet, U. Expressing personality through non-verbal Behaviour in Real-Time Interaction. *Front. Psychol.* **12**, 5474. 10.3389/fpsyg.2021.660895 (2021). 10.3389/fpsyg.2021.660895 PMC8662698 34899452

[47] Rickel, J. et al. Toward a New Generation of virtual humans for interactive experiences. *IEEE Intell. Syst.* **17** (4), 32–38. 10.1109/MIS.2002.1024750 (2002).

[48] Laranjo, L. et al. Conversational agents in healthcare: A systematic review, *Journal of the American Medical Informatics Association*, vol. 25, no. 9. Oxford University Press, pp. 1248–1258, doi: (2018). 10.1093/jamia/ocy072 10.1093/jamia/ocy072 PMC6118869 30010941

[49] Dar, S., Ekart, A. & Bernardet, U. The virtual human breathing coach, pp. 434–436, doi: (2022). 10.1109/vrw55335.2022.00095

[50] Bian, Y. et al. Effects of pedagogical agent’s personality and emotional feedback strategy on Chinese students’ learning experiences and performance: a study based on virtual Tai Chi training studio. *Conf. Hum. Factors Comput. Syst. - Proc.* 433–444. 10.1145/2858036.2858351 (2016).

[51] Hudlicka, E. Virtual training and coaching of Health Behavior: Example from Mindfulness Meditation Training. *Patient Educ. Couns.* **92** (2), 160–166. 10.1016/j.pec.2013.05.007 (2013). 23809167 10.1016/j.pec.2013.05.007 PMC3970714

[52] DMello, S. & Graesser, A. AutoTutor and affective autotutor: learning by talking with cognitively and emotionally intelligent computers that talk back. *ACM Trans. Interact. Intell. Syst.* **2** (4). 10.1145/2395123.2395128 (2012).

[53] Shamekhi, A., Bickmore, T., Lestoquoy, A. & Gardiner, P. Augmenting group medical visits with conversational agents for stress management behavior change, in *Lecture Notes in Computer Science (including subseries Lecture Notes in Artificial Intelligence and Lecture Notes in Bioinformatics)*, vol. 10171 LNCS, pp. 55–67, doi: (2017). 10.1007/978-3-319-55134-0_5

[54] S. Dar, V. Lush and U. Bernardet, "The Virtual Human Breathing Relaxation System," 2019 5th Experiment International Conference (exp.at’19), Funchal, Portugal, pp. 276-277, 10.1109/EXPAT.2019.8876478. (2019).

[55] Perera, A. Uncanny Valley: Examples, Effects And Theory, *Cogn. Psychol.*, pp. 1–21, [Online]. Available: (2023). https://www.simplypsychology.org/uncanny-valley.html#

[56] Marr, B. The Uncanny Valley: Advancements And Anxieties Of AI That Mimics Life, (2024). https://www.forbes.com/sites/bernardmarr/2024/02/07/the-uncanny-valley-advancements-and-anxieties-of-ai-that-mimics-life/ (accessed Sep. 12, 2024).

[57] Kang, J. & Wei, L. Promises of anthropomorphism in virtual coaches: current research and future directions. *PervasiveHealth Pervasive Comput. Technol. Healthc.* 243–246. 10.1145/3421937.3421945 (2020).

[58] Zhong, V. J., Murset, N., Jager, J. & Schmiedel, T. Exploring Variables That Affect Robot Likeability, *ACM/IEEE Int. Conf. Human-Robot Interact.*, vol. 2022-March, pp. 1140–1145, doi: (2022). 10.1109/HRI53351.2022.9889602

[59] Ferstl, Y., Thomas, S., Guiard, C., Ennis, C. & McDonnell, R. Human or Robot? Investigating voice, appearance and gesture motion realism of conversational social agents, in *Proceedings of the 21st ACM International Conference on Intelligent Virtual Agents, IVA* 2021, pp. 76–83, doi: (2021). 10.1145/3472306.3478338

[60] Byom, L. J. & Mutlu, B. Theory of mind: mechanisms, methods, and new directions. *Front. Hum. Neurosci.* **7**, 413. 10.3389/fnhum.2013.00413 (2013). 23964218 10.3389/fnhum.2013.00413 PMC3737477

[61] Knapp, M. L., Hall, J. A. & Horgan, T. G. *Nonverbal Communication in Human Interaction* 8th edn (Monica Eckman, 2012).

[62] Gratch, J. *et al.* Can Virtual Humans Be More Engaging Than Real Ones?. In: Jacko, J.A. (eds) Human-Computer Interaction. HCI Intelligent Multimodal Interaction Environments. HCI 2007. Lecture Notes in Computer Science, vol 4552. Springer, Berlin, Heidelberg. 10.1007/978-3-540-73110-8_30 (2007).

[63] Huang, Lixing, Louis-Philippe Morency and Jonathan Gratch. “Virtual Rapport 2.0.”* International Conference on Intelligent Virtual Agents (2011)*. pp. 68–79, doi: 10.1007/978-3-642-23974-8_8

[64] Ranjbartabar, H., Richards, D., Bilgin, A. A. & Kutay, C. First impressions count! The role of the Human’s emotional state on Rapport established with an empathic versus neutral virtual therapist. *IEEE Trans. Affect. Comput.* **12** (3), 788–800. 10.1109/TAFFC.2019.2899305 (2021).

[65] Https://unity.com, Unity3D.

[66] Mixamo. Available at: https://www.mixamo.com/ (Accessed: 13 December 2024).

[67] Games, H. playMaker.

[68] Box breathing: Getting started with box breathing, how to do it, benefits and tips (April 30, 2023) WebMD. Available at: https://www.webmd.com/balance/what-is-box-breathing (Accessed: 13 December 2024).

[69] Breathing exercises for stress, (2022) NHS choices. Available at: https://www.nhs.uk/mental-health/self-help/guides-tools-and-activities/breathing-exercises-for-stress/ (Accessed: 13 December 2024).

[70] Prajapati, Dr.V.C. (2024) What is box breathing?, What is Box Breathing? Available at: https://www.icliniq.com/articles/respiratory-health/box-breathing-a-walkthrough (Accessed: 13 December 2024).

[71] Sheldon, R. B. *Clinical Methods: The History, Physical, and Laboratory Examinations*, 3rd ed. (1990). 21250045

[72] Lewis, J. R. The System Usability Scale: past, Present, and Future. *Int. J. Hum. Comput. Interact.* **34**, 577–590. 10.1080/10447318.2018.1455307 (2018).

[73] Team, R. C. *R: A Language and Environment for Statistical Computing* (R Foundation for Statistical Computing, 2013).

[74] Zibrek, K., Martin, S. & McDonnell, R. Is photorealism important for perception of expressive virtual humans in virtual reality? *ACM Trans. Appl. Percept.* **16** (3). 10.1145/3349609 (2019).

[75] Karuzaki, E. et al. Realistic virtual humans for cultural heritage applications. *Heritage* **4** (4), 4148–4171. 10.3390/heritage4040228 (2021).

[76] Dar, S. & Bernardet, U. When agents become partners: A review of the role the implicit plays in the interaction with artificial social agents, *Multimodal Technologies and Interaction*, vol. 4, no. 4. MDPI AG, pp. 1–24, doi: (2020). 10.3390/mti4040081

[77] Parmar, D., Olafsson, S., Utami, D. & Bickmore, T. Looking the part: The effect of attire and setting on perceptions of a virtual health counselor, *Proc. 18th Int. Conf. Intell. Virtual Agents, IVA* pp. 301–306, 2018, doi: (2018). 10.1145/3267851.3267915

