# Supplementary material for: Usability, acceptance, and the role of realism in virtual humans for breathing exercise training
Source: Sci Rep. 2025 Jan 9;15:1536. doi: 10.1038/s41598-024-82886-7 (PMC11718229; doi:10.1038/s41598-024-82886-7)
Supplement: Supplementary file 1 — Supplementary Material 1 [file 41598_2024_82886_MOESM1_ESM.pdf]

Usability, Acceptance, and the Role of Realism in Virtual Humans for Breathing Exercise Training

Sanobar Dar, Aniko Ekart and Ulysses Bernardet

Supplementary material

The questions derived from the System Usability Scale (SUS) are marked with *

A. POST EXERCISE QUESTIONS

	QUESTION	Scale Min	Scale Max
1.	How did you find the breathing exercise?	Easy	Difficult
2.	How did you find the breathing exercise?	Boring	Interesting
3.	How did you find the breathing exercise?	Too Short	Too Long
4.	How easy was it to follow the coach?	Easy	Difficult
5.	How is your current breathing rate compared to that of the start of experiment?	Slower	Faster
6.	After doing this exercise how relaxed do you feel?	Not At All	Very Much
7.	Did the coach help you to relax?	Not At All	Very Much
8.	Did the coach influence your breathing?	Not At All	Very Much
9.	How much did the coach's instructions influence your breathing?	Not At All	Very Much
10.	How much did the coach's breathing movements influence your breathing?	Not At All	Very Much

B. POST EXPERIMENT QUESTIONS

	QUESTION	Scale Min	Scale Max
1.	How did you find the breathing exercise?	Easy	Difficult
2.	How did you find the breathing exercise?	Boring	Interesting
3.	How did you find the breathing exercise?	Too Short	Too Long

4.	How would you rate the coach?	Very Likeable	Not Likeable At All
5.	How would you rate the coach?	Very Trustworthy	Not Trustworthy At All
6.	How is your current breathing rate compared to that of the start of experiment?	Slower	Faster
7.	After doing this exercise how relaxed do you feel	Not At All	Very Much
8.	Did the coach help you to relax?	Not At All	Very Much
9.	Did the coach influence your breathing?	Not At All	Very Much
10.	How much did the coach's instructions influence your breathing?	Not At All	Very Much
11.	How much did the coach's breathing movements influence your breathing?	Not At All	Very Much
12.	How easy was it to follow the coach?	Easy	Difficult
13.	Coach Kim is a computer-generated human. How realistic did you find her and the following aspects?	Very Realistic	Not Realistic At All
14.	Breathing	Very Realistic	Not Realistic At All
15.	Voice	Very Realistic	Not Realistic At All
16.	Gestures	Very Realistic	Not Realistic At All
17.	Movement	Very Realistic	Not Realistic At All
18.	How certain are you that the virtual coach will be able to replace the human coach?	Not At All	Very Much
19.	*How well do you think did the system run?	Not At All	Very Well
20.	*How much would you be interested in continuing using the system?	Not At All	Very much
21.	Do you have any further comments or feedback?		
